# Lipoprotein(a) Testing Trends in the United States 2015-2024: An Analysis of 300 Million Individuals

**DOI:** 10.1016/j.jacadv.2025.102205

**Published:** 2025-09-23

**Authors:** Mattheus Ramsis, Mustafa Naguib, Marissa Dzotsi, Michael Wilkinson, Ehtisham Mahmud, Pam Taub, Harpreet S. Bhatia

**Affiliations:** Division of Cardiology, Department of Medicine, University of California-San Diego, San Diego, California, USA

**Keywords:** bioinformatics, electronic health record, epidemiology, lipids, lipoprotein (a), prevention, United States

**Figure:**
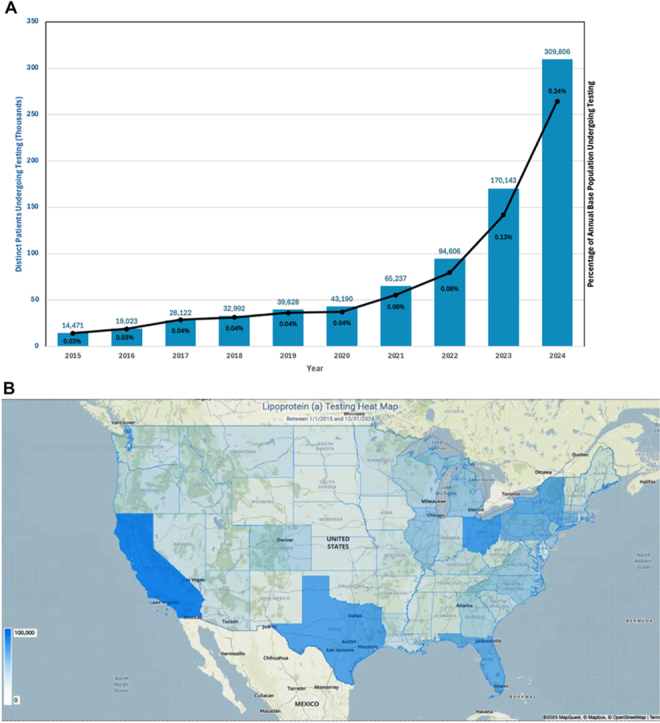

Lipoprotein(a) [Lp(a)] is a genetically determined lipoprotein associated with increased risk for cardiovascular disease, including myocardial infarction, stroke, and aortic stenosis.^1^ Elevated Lp(a) is common, with approximately 1 in 5 individuals having Lp(a) >50 mg/dL (>125 nmol/L). However, Lp(a) testing remains underutilized in clinical practice, and data on national testing trends are limited.^2^^,^^3^ This study aimed to assess trends in Lp(a) testing across the United States from 2015 to 2024 using a large, real-world data set.

## Methods

We conducted a retrospective analysis of Lp(a) testing rates across the United States from January 1, 2015, to December 31, 2024. Data used in this study came from Epic Cosmos, a data set created in collaboration with a community of Epic health systems representing more than 300 million patient records from over 1715 hospitals and 41,000 clinics from all 50 states, D.C., Lebanon, and Saudi Arabia. The current count values for patients, hospitals, and clinics are available on Epic Cosmos website.^4^ Although the Epic Cosmos data dictionary includes Lebanon and Saudi Arabia as standardized site locations, no patients from these countries were present in our analytic cohort; thus, all analyses were restricted to individuals within the United States. Testing rates were calculated as the number of distinct patients tested per year and as the testing rate per annual patient population. Geographical variation was visualized using a heat map of testing by state. All data are deidentified in compliance with Health Insurance Portability and Accountability Act standards and governed under Epic’s “Rules of the Road” for institutional data use.

**What is the clinical question being addressed?**

What are the national trends in lipoprotein(a) [Lp(a)] testing in the United States?

**What is the main finding?**

Lp(a) testing remains rare but has increased modestly in recent years, with substantial demographic and geographic variability.

## Results

**Figure 1:**
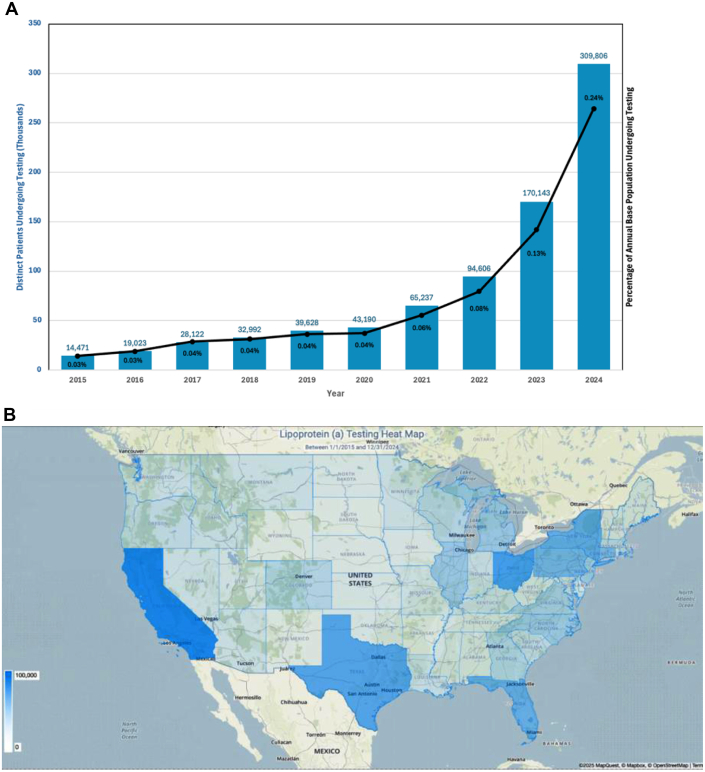
**National Trends and Regional Distribution of Lipoprotein(a) Testing in the United States (2015-2024)** (A) Yearly trends in distinct Lp(a) testing in the United States, showing a substantial increase from 2015 to 2024. (B) Geographic distribution of Lp(a) testing, highlighting regional variation in testing uptake. Created using Epic Cosmos. Analysis performed by Mattheus Ramsis, MD, and internally reviewed by Mattheus Ramsis, MD, at UCSD Health on 04/17/25. Lp(a) = lipoprotein(a).

From 2015 to 2024, a total of 728,550 (0.2% of U.S. population) distinct patients underwent Lp(a) testing. Over this period, the annual number of tested patients increased significantly from 14,471 in 2015 to 309,806 in 2024 (chi-square test, *P* < 0.001) (Figure 1A). The annual percentage of the total patient population undergoing Lp(a) testing also increased, rising from 0.03% in 2015 to 0.24% in 2024. Geographical analysis revealed considerable regional variation, with the highest number of tests performed in California (11.6%), Ohio (8.6%), and Texas (7.6%) (Figure 1B). Age-stratified analyses revealed that testing was most frequently conducted among adults between the ages of 50 and 65 years (n = 253,409; 34.8%). Lp(a) testing rates were similar by sex, with 51.8% (n = 377,053) among males and 48.2% (n = 351,384) among females. Among patients undergoing testing with reported ethnic identity, only 51,950 (7.1%) identified as Hispanic or Latino, while the remaining 676,600 (92.9%) identified as non-Hispanic. Most patients undergoing testing with reported racial data identified as White (n = 581,101; 79.8%), followed by other race (n = 82,320; 11.3%), Black or African American (n = 65,683; 9.0%), Asian (n = 44,131; 6.1%), and fewer non-Hispanic individuals identifying as American Indian or Alaska Native or selecting “none of the above.” Over the past decade, Lp(a) testing in the United States has progressively shifted from predominantly mass-based assays (mg/dL) to molar assays (nmol/L), with molar assays accounting for 64.2% of all Lp(a) tests by 2024.

## Discussion

The observed increase in Lp(a) testing over the past decade likely reflects growing awareness of its role in cardiovascular risk stratification. However, persistently low rates of testing and the variation by region, age, and race/ethnicity suggest that further efforts are needed to promote standardized Lp(a) screening guidelines nationwide.^5^ In line with current recommendations for Lp(a) testing, we observed a favorable shift in practice from using mass assays to the preferred molar assay.^6^ These findings highlight the potential for targeted educational and policy interventions to reduce disparities in cardiovascular risk assessment.

## Conclusions

This study demonstrates a substantial increase in Lp(a) testing across the United States over the past decade, but with persistently low rates of testing with significant regional and demographic variation. Further research is needed to understand the drivers of these trends and to optimize the use of Lp(a) testing for cardiovascular risk stratification.

## Funding support and author disclosures

Dr Ramsis is supported by the 10.13039/100005485American College of Cardiology Foundation Executive Leadership Award, the 10.13039/100001009Robert A. Winn Career Development Award, and the 10.13039/100000133Agency for Healthcare Research and Quality (AHRQ) Dr Taub is supported by 10.13039/100000002NIH grant 2R01DK118278-06A1 and has served as a consultant to 10.13039/100002429Amgen, 10.13039/100001003Boehringer Ingelheim, 10.13039/100004312Lilly, 10.13039/501100004191Novo Nordisk, 10.13039/100004336Novartis, 10.13039/100004374Medtronic, Jazz, 10.13039/100004326Bayer, 10.13039/100014931Arrowhead, and 10.13039/100004337Roche. Dr Bhatia is supported by 10.13039/100000002NIH grant 1K08HL166962 and has served as a consultant/advisor for Kaneka, 10.13039/100004336Novartis, NewAmsterdam, 10.13039/100014931Arrowhead, and 10.13039/100000046Abbott. Dr Wilkinson is supported by 10.13039/100002429Amgen; has served as a consultant to Amarin, 10.13039/100009857Regeneron, The Kinetix Group, Kaneka, Ionis, NewAmsterdam, and 10.13039/100004336Novartis (payments made to institution); is a member of advisory boards for 10.13039/100004336Novartis, NewAmsterdam, and 10.13039/100009857Regeneron; and has received contracted research support (to his institution) from 10.13039/100002429Amgen, 10.13039/100004336Novartis, Ionis, Mineralys, 10.13039/100004312Lilly, and Silence. All other authors have reported that they have no relationships relevant to the contents of this paper to disclose. This research was supported by the UC San Diego BEACON Center for Health Data Science.
